# Don’t black box your research

**DOI:** 10.1016/j.patter.2026.101663

**Published:** 2026-09-11

**Authors:** Alejandra Alvarado, Andrew L. Hufton

**Affiliations:** Scientific editor, *Patterns*; Editor-in-chief, *Patterns*

## Main text

As generative AI tools become routine parts of research, the journal has growing concerns about how this transformation will affect the reproducibility of research. We call upon our authors to take steps to ensure that others will be able to reproduce their work now and many years into the future. Don’t let your research become a black box.

In a tutorial published in this month’s issue of *Patterns*, Dunn et al. provide guidance for researchers grappling with this challenging issue. They focus specifically on research that uses large language models (LLMs), but much of their advice can be generalized to any study that relies on AI models.

They first introduce a question: is the LLM “on” or “off” the data path. They write, “An LLM is *on* the data path when data move through it at run time, for example when the LLM performs image segmentation or classification. An LLM is *off* the data path when it is used to produce an artifact that sits on the path, for example when it writes code that is then committed to the pipeline.” When the LLM is off the data path, they argue that careful validation and subsequent archiving of the outputs from the model (e.g., the analysis code) may be sufficient for ensuring reproducibility. If the LLM is on the data path, ensuring reproducibility is more challenging. If the LLM must be on the data path, they recommend recording model versions, quantifying model determinism and inter-model agreement, and using, where possible, open-weights models.

Importantly, Dunn et al. note that whether an LLM is on or off path can depend on the paper’s context. We argue that journal context also matters. At *Patterns,* our editorial interest will naturally tend to focus on the AI-powered methods and workflows in a paper. We may therefore be more likely to perceive an AI model as “on path.”

Reproducibility concerns are starkest in cases where proprietary AI models are used “on the data path.” Since proprietary, closed-weights models are not publicly archived, there is no guarantee that future researchers will be able to replicate an analysis with the same model version. This and the rapid evolution of commercial AI services can make it challenging for others to critically reanalyze a published work or workflow that relies on closed-source AI models. Moreover, if the training datasets for a model are not disclosed, controlling for issues like data leakage or critically assessing potential bias in model training can be more challenging. Further imperiling transparency and reproducibility, a range of AI-powered products targeting researchers now exist that do not declare the underlying model or model versions.

For all these reasons, we strongly prefer research based on open-source models or, at minimum, works that have been replicated with at least one open-weights model (Wang et al., 2023; *Patterns*). At the same time, we do not want to exclude exciting, innovative work from the journal. Striking a balance here remains challenging. We will continue to be particularly flexible with papers that are studying the social or societal impact of AI technologies. See, for example, these two *Patterns* papers: Liang et al., 2023, and Liang et al., 2025. We are aware that asking authors to reproduce these kinds of studies with open-source models may be impossible or may answer a different question entirely.

Regardless of how LLMs or other generative AI tools are used, transparency is key. We ask authors to declare any such use in the methods section of their paper or in the dedicated AI use declaration statement. We invite our authors to check the latest Cell Press and Elsevier policies on generative AI and AI-assisted technologies in the manuscript preparation process before submitting to the journal. Notably, we do now allow limited use of AI tools in the preparation of certain explanatory figures, but we require that this be declared transparently in the paper.

*Patterns* is dedicated to promoting open and reproducible data science; thus, we believe reproducibility, transparency, and openness should not be bureaucratic hurdles but central aspects of how we judge scientific excellence. We invite our authors to think creatively and critically about how to make their AI-powered research as open and reproducible as possible. If you are unsure whether your use of AI meets our standards, please do reach out. We would rather help you get it right and avoid the black box than reject an otherwise strong paper.

